# Post-Voltage-Boost Circuit-Supported Single-Ended Class-B Amplifier for Piezoelectric Transducer Applications

**DOI:** 10.3390/s20185412

**Published:** 2020-09-21

**Authors:** Jungsuk Kim, Kiheum You, Hojong Choi

**Affiliations:** 1 Department of Biomedical Engineering, Gachon University, 191 Hambakmoe-ro, Yeonsu-Gu, Incheon 21936, Korea; jungsuk@bme.gachon.ac.kr; 2 Department of Medical IT Convergence Engineering, Kumoh National Institute of Technology, 350-27 Gumi-daero, Gumi 39253, Korea; rlgma12@kumoh.ac.kr

**Keywords:** amplifier, piezoelectric transducer, post-voltage-boost circuit

## Abstract

Piezoelectric transducers are important devices that are triggered by amplifier circuits in mobile ultrasound systems. Therefore, amplifier performance is vital because it determines the acoustic piezoelectric transducer performances. Particularly, mobile ultrasound applications have strict battery performance and current consumption requirements; hence, amplifier devices should exhibit good efficiency because the direct current (DC) voltage in the battery are provided to the supply voltages of the amplifier, thus limiting the maximum DC drain voltages of the main transistors in the amplifier. The maximum DC drain voltages are related with maximum output power if the choke inductor in the amplifier is used. Therefore, a need to improve the amplifier performance of piezoelectric transducers exists for mobile ultrasound applications. In this study, a post-voltage-boost circuit-supported class-B amplifier used for mobile ultrasound applications was developed to increase the acoustic performance of piezoelectric transducers. The measured voltage of the post-voltage-boost circuit-supported class-B amplifier (62 V_P-P_) is higher than that of only a class-B amplifier (50 V_P-P_) at 15 MHz and 100 mV_P-P_ input. By performing the pulse-echo measurement test, the echo signal with the post-voltage-boost circuit-supported class-B amplifier (10.39 mV_P-P_) was also noted to be higher than that with only a class-B amplifier (6.15 mV_P-P_). Therefore, this designed post-voltage-boost circuit can help improve the acoustic amplitude of piezoelectric transducers used for mobile ultrasound applications.

## 1. Introduction

Piezoelectric transducers have been widely utilized for a variety of ultrasound components, such as touch-pad smartphone and parking assistance sensors, pulse-echo measurement instruments, nondestructive testing systems, submarine radar devices, material characterization systems, and acoustic trapping devices [1,2,3,4,5]. The transducers generate acoustic waves triggered by electrical power or waves stimulated by acoustic forces [6]. The primary capacitance with parasitic resistance, capacitance, and inductance in the transducer equivalent circuit model represent non-linear characteristics because the transducer is sensitive to voltage and frequency variances [7]. The maximum alternative current (AC) applied voltages to the piezoelectric material must be less than maximum DC applied voltages to the piezoelectric material [8,9]. Within limited DC applied voltages, higher applied voltages could produce higher acoustic amplitude generated from the transducer [10,11]. The applied voltage with different frequencies ranging between resonant and anti-resonant frequencies, owing to unmatched impedance conditions, could generate lower or very weak acoustic waves [8]. As a result, ensuring the proper design of electronic devices is key to attaining piezoelectric transducers with stable performance. In electronic devices, amplifiers are among the electronic devices that most significantly affect the sensitivity performance of ultrasound systems [12]. They are also considered final-stage electronics, with the exception of expander circuits, which are required for piezoelectric transducers with high voltage amplitude waveform [13]. In ultrasound applications, the amplifier is usually employed to amplify a variety of pure sine, square, or coded signals depending on the ultrasound imaging modes [14,15,16]. These signals are typically generated from digital signal processing or field-programmable gate array (FPGA) electronics, after which they are transmitted to piezoelectric transducers to produce sensitive vibrational waves [17].

Currently, the applications of mobile ultrasound are increasing in the fields of touch-pad smartphones and mobile medical instruments [18,19]. However, these systems are normally required to have miniaturized piezoelectric transducers compared to large bench-top systems [8,18,20,21,22]. Therefore, some small-size piezoelectric transducers have limited maximum direct current (DC) applied voltages. For 4 × 4 × 4-mm^3^ PMN-PZT piezoelectric material, the measured maximum DC applied voltages are approximately 150 V_P-P_ [12,23]. The maximum applied DC voltage restricts the maximum alternating current (AC) voltages for amplifiers in mobile systems [24,25,26]. Thus, maximum AC voltages that are less than DC voltages can directly trigger the piezoelectric transducers. The amplifiers with higher AC voltages are more useful, especially for mobile ultrasound systems [27]. In fact, ultrasound companies tried to apply much lower AC voltages to reduce heat effects to the transducers, thus reducing the sensitivity performances. The amplifiers with higher AC voltages and efficiency are more desirable. For example, in mobile ultrasound systems, piezoelectric array-type transducers that are smaller than 1 × 1 × 1 mm^3^ can be utilized. For most mobile ultrasound systems owing to space limitations, the smaller size piezoelectric transducers can be utilized [28,29]. Therefore, amplifier performance improvement in mobile ultrasound systems is beneficial to produce high acoustic amplitudes.

Several electronics research works have focused on improving the amplifier performance for piezoelectric transducer applications. The Texas Instruments medical device group developed a pre-distortion technique using analog-to-digital converters (ADCs), digital-to-analog-converters (DACs), memory, and FPGA electronics for ultrasound applications [30]. This scheme is useful for a single ultrasonic transducer and class-A amplifier, which are required to possess high power consumption in the bench-top ultrasound machines. The pre-linearizer is supposed to be placed before the amplifier circuit [31,32,33]. However, the post-distortion linearizer circuit is located between the amplifier and piezoelectric transducer or between the expander after the amplifier and piezoelectric transducer [34,35]. Likewise, using a similar circuit type after the amplifier could easily enable the controlled performance of the piezoelectric transducer because this circuit is located between the amplifier and transducer. Therefore, the placement of the circuit before piezoelectric transducers may be more useful because they have some unwanted parasitic capacitance, inductance, and resistance, such that similar circuit types could be further customized between the amplifier and piezoelectric transducers. A post-distortion linearizer circuit was developed for a class-A amplifier and intended for bench-top ultrasound instrument applications [34]. This approach helps achieve a flatter gain within wide input power ranges for a class-A power amplifier required to have high power consumption.

However, our proposed scheme is intended to increase the voltage output for a class-B amplifier, thus, improving the acoustic waveform amplitude for mobile ultrasound systems with limited DC voltages. Nonlinear amplifiers such as the class-B amplifier are preferable for mobile ultrasound instruments owing to their lower DC power consumption [19]. For typical ultrasound applications, a single-ended-type class-B amplifier is preferable compared to a differential-ended amplifier for piezoelectric transducers, because some piezoelectric transducers have large undesirable capacitances in their equivalent circuit models [36,37,38]. A differential-ended amplifier scheme can change the resonant frequency of piezoelectric transducers because the change from a differential path to a single path requires the use of large inductive-type transformers [39,40]. In addition, mobile systems have limited DC power consumption owing to their battery capacity, such that electronic device performance is also affected when designing transducer devices [8,41,42,43].

**Figure 1:**
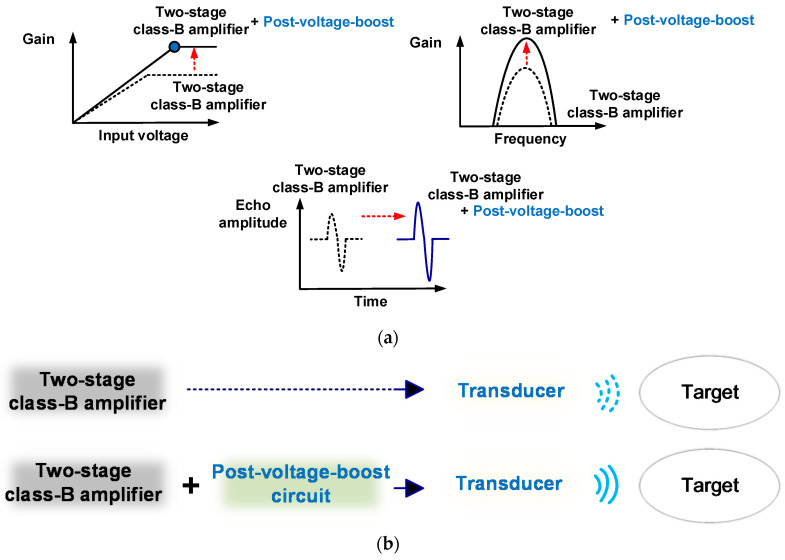
(**a**) Performance concepts of the proposed scheme; (**b**) single-ended class-B amplifier with and without a post-voltage-boost circuit.

In this study, we first implemented a post-voltage-boost circuit-supported by a single-ended class-B amplifier for piezoelectric transducers. Figure 1 shows the concept of the proposed post-voltage-boost circuit for the class-B amplifier and piezoelectric transducer applications. As shown in Figure 1a,b, the post-voltage-boost circuit is located between the class-B amplifier and piezoelectric transducer, enabling the performance control of the class-B amplifier and piezoelectric transducer. Therefore, the output voltage of the post-voltage-boost circuit-supported class-B amplifier can be varied such that the echo signal amplitude of the piezoelectric transducer can be affected as well, as shown in Figure 1a.

Section 2 describes the operational mechanism, circuit schematic diagram, and mathematical analysis of the designed post-voltage-boost circuit and single-ended class-B amplifier. Section 3 demonstrates the improved electrical and acoustic performance of the designed electronic devices using a piezoelectric transducer with respect to the electronic measurement. Finally, Section 4 concludes the paper.

## 2. Materials and Methods

There are two different types of ultrasonic transducers, namely the capacitive micromachined ultrasonic transducer (CMUT), which is driven by a current signal, and the piezoelectric transducer, which is driven by a voltage signal [4]. A piezoelectric transducer was used for this research. The high voltage signal output from the amplifier is transmitted to the piezoelectric transducer [44,45]. Higher output voltage amplitude from the amplifier is transmitted to obtain higher acoustic signal amplitude generated from the piezoelectric transducer [28,46,47]. Thus, a stronger acoustic signal can be achieved from piezoelectric transducers [8,12]. Therefore, a post-voltage-boost circuit was designed to obtain a higher acoustic signal from piezoelectric transducers. The output signal of a single-ended class-B amplifier is transferred to the input of the post-voltage-boost circuit. The received signal from the amplifier is modulated in the post-voltage-boost circuit.

### 2.1. Schematic of Class-B Amplifier and Post-Voltage-Boost Circuit

**Figure 2:**
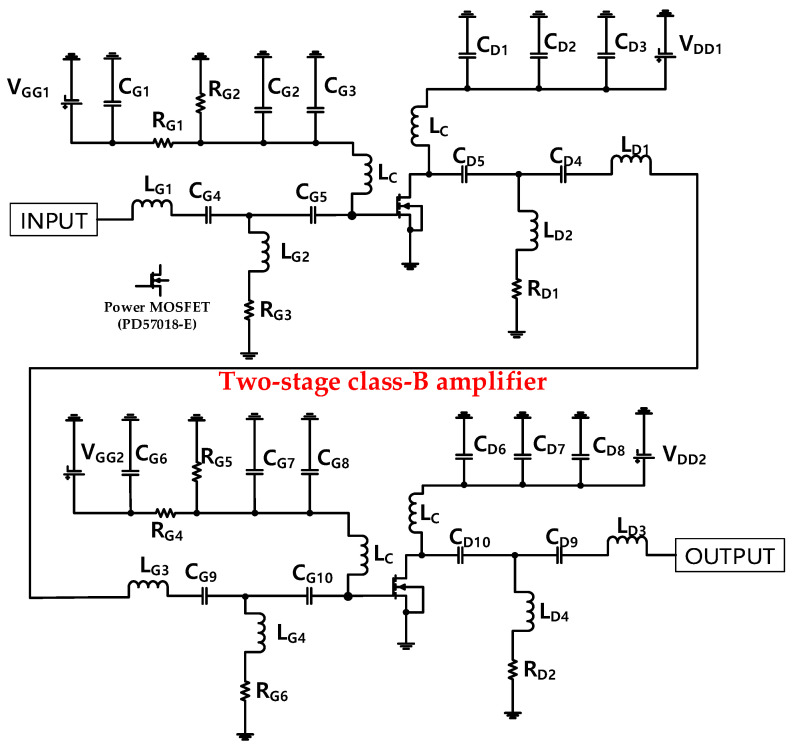
Schematic of the single-ended class-B amplifier.

In Figure 2, a schematic of a single-ended two-stage class-B amplifier with a post-voltage-boost circuit shows the operational mechanisms. In this amplifier, a power metal-oxide-semiconductor field-effect transistor (power MOSFET, PD57018-E, STMicroelectronics Corp., Geneva, Switzerland) was used as the main transistor. The first- and second-stage amplifiers were applied with a 3.1-V gate bias voltage to be operated in the class-B mode. The inductors at the gate and drain sides are choke inductors (L_C_ = 1 μH), which could minimize the voltage drop when DC bias voltages were used [48,49,50]. In addition, the electrolytic capacitors (C_G1_, C_D3_, C_G6_, and C_D8_ = 220 μF) and ceramic capacitors (C_G2_, C_D2_, C_G7_, and C_D7_ = 1000 pF, C_G3_, C_D1_, C_G8_, and C_D6_ = 47 pF) were used to minimize the noise signals from the DC power supply [51,52]. The input inductors, capacitors, and resistors (L_G1_ and L_G3_ = 22 nH, C_G4_ and C_G9_ = 560 pF, C_G5_ and C_G10_ = 330 pF, L_G2_ and L_G4_ = 1000 nH, and R_G3_ and R_G6_ = 200 Ω), and output inductors, capacitors, and resistors (L_D1_ and L_D3_ = 120 nH, C_D4_ and C_D9_ = 330 pF, C_D5_ and C_D10_ = 820 pF, L_D2_ and L_D4_ = 500 nH, and R_D1_ and R_D2_ = 200 Ω) in the first- and second-stage class-B amplifiers were configured for 50 Ω impedance matching conditions at a center frequency of 15 MHz.

**Figure 3:**
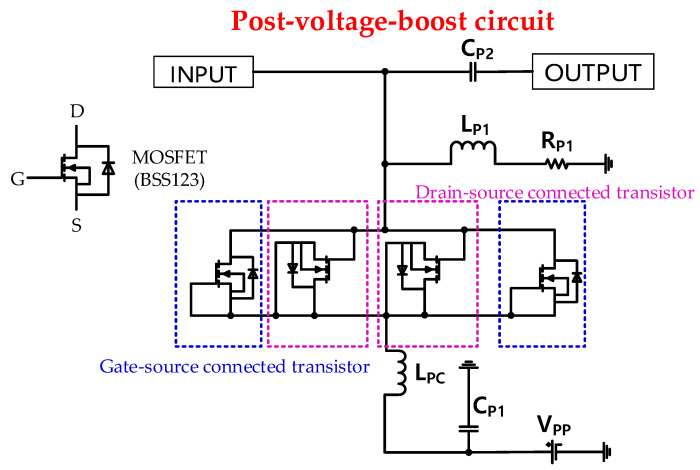
Schematic of the post-voltage-boost circuit.

Figure 3 shows a schematic of the post-voltage-boost circuit. The post-voltage-boost circuit input (INPUT) is connected from the amplifier output (OUTPUT), as shown in Figure 2. Thus, the output signal of the amplifier (OUTPUT) is transmitted to the input (INPUT) of the post-voltage-boost circuit, as shown in Figure 3. The post-voltage-boost circuit consists of a DC bias signal input (V_PP_), electrolytic capacitor (C_P1_ = 220 μF), choke inductor (L_PC_ = 1 μH), four transistors, inductor (L_P1_ = 2.2 μH), resistor (R_P1_ = 50 Ω), and capacitor (C_P2_ = 220 pF). In the post-voltage-boost circuit, the four transistors are the gate-source or drain-source connected type. Gate-source connected transistors can be interpreted as variable resistors, and drain-source connected transistors can be interpreted as variable capacitors in the equivalent circuit model [24,41]. The gate-source or drain-source connected transistors behave as variable resistors or capacitors depending on the applied bias DC voltage of the post-voltage-boost circuit. Because the designed class-B amplifier is operated under a high-voltage and high-current environment, it is designed to be stable in that environment using four MOSFETs in a post-voltage-boost circuit. The four MOSFETs were selected due to high voltage and high current environment. Therefore, in the theoretical approaches, the parasitic resistance of four MOSFETs are reduced by ¼ times of one MOSFET, but the parasitic capacitance of four MOSFETs are increased by 4 times of one MOSFET. Therefore, the variable capacitors and resistors, which depend on the applied DC bias voltages (V_PP_), could offset the variances of the inductors (L_D3_ and L_D4_) and capacitors (C_D10_ and C_D9_), including the large unwanted parasitic capacitances of the power MOSFET in the second-stage amplifier. In addition, unnecessary harmonic signals that are generated from the amplifier can be reduced using a resonance filter structure in the post-voltage-boost circuit (C_P2_, L_P1_, and R_P1_) [53,54,55,56].

### 2.2. Equivalent Circuit Analysis of Class-B Amplifier

A two-stage class-B amplifier can be estimated by performing an equivalent circuit analysis. To simplify the equivalent circuit analysis, the internal resistance and inductance values of the transistors at each stage (gate, drain, and source) were not considered in the large-signal nonlinear power MOSFET model [24,29,57,58].

**Figure 4:**
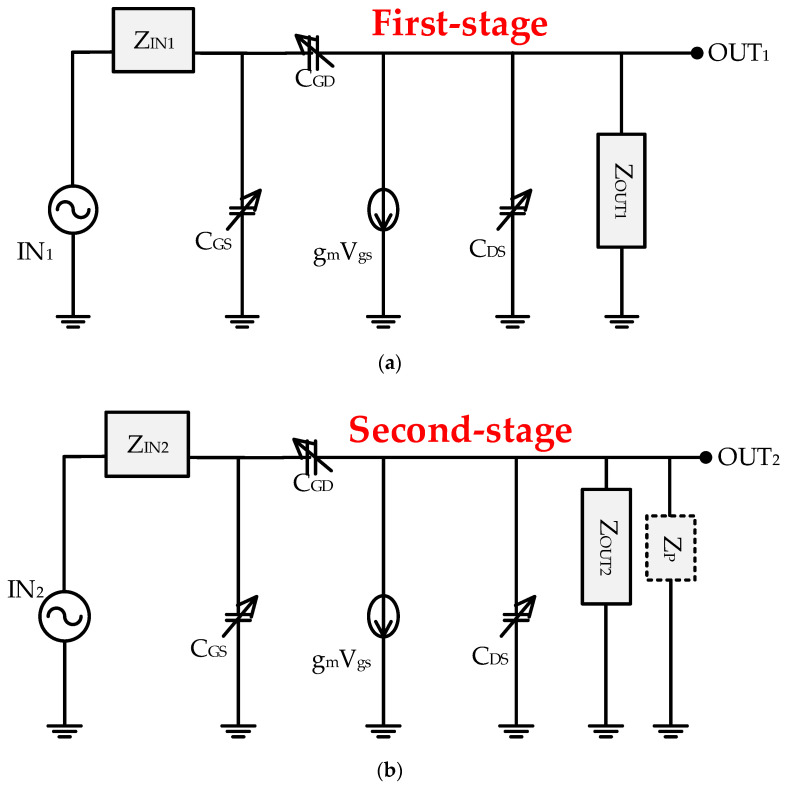
(**a**) First-stage equivalent circuit of the amplifier; (**b**) second-stage equivalent circuit of the amplifier.

Figure 4a,b show the simplified equivalent circuit models of the first and second stages of the class-B amplifier, respectively. IN_1_ and IN_2_ represent the signal inputs, and OUT_1_ and OUT_2_ represent the signal outputs of the two-stage class-B amplifier. Z_IN1_ and Z_IN2_ are the impedance values observed at the signal’s input, and Z_OUT1_ and Z_OUT2_ are the impedance values observed at the output of the signal [59]. C_GS_ represent the transistor’s internal gate-source capacitances. The capacitances (C_GD_, C_GS_, and C_DS_) represent the transistor’s internal parasitic capacitances [60]. Each internal capacitance has variable values according to the applied gate-source and gate-drain voltage. The parameters g_m_ represents the transconductance values of the transistors.

The impedance, input and output poles, and final output voltage of the amplifiers are obtained using the equivalent circuit analysis in Figure 4a,b. The input impedance of the first-stage amplifier (Z_IN1_), shown in Figure 4a, is obtained from the circuit components, shown in Figure 2, as follows: Z_IN1_ is the impedance observed from the signal input in the first-stage amplifier [61]. From the signal input, the inductor and capacitor (L_G1_ and C_G4_) are parallel to the inductor and resistor (L_G2_ and R_G3_) and capacitor (C_G5_). In addition, the output impedance of the first-stage amplifier (Z_OUT1_) represented in Figure 4a is obtained from the circuit components in Figure 2 as follows: Z_OUT1_ is the impedance observed from the signal output in the first-stage amplifier [53]. From the signal output, the inductor and capacitor (L_D1_ and C_D4_) are parallel to the inductor and resistor (L_D2_ and R_D1_) and capacitor (C_D5_). Z_IN1_ and Z_OUT1_ of the first stage are expressed by Equation (1).

(1) $$\begin{array}{c} Z_{IN1}=(j2\pi fL_{G1}+\frac{1}{j2\pi fC_{G4}})+\left\{(j2\pi fL_{G2}+R_{G3})∥\frac{1}{j2\pi fC_{G5}}\right\},\;\\ Z_{OUT1}=(j2\pi fL_{D1}+\frac{1}{j2\pi fC_{D4}})+\left\{(j2\pi fL_{D2}+R_{D1})∥\frac{1}{j2\pi fC_{D5}}\right\}, \end{array}$$

where f of j2*π*f is the center frequency of the amplifier.

The input impedance of the second-stage amplifier (Z_IN2_) illustrated in Figure 4b is obtained from the circuit components shown in Figure 2 as follows: Z_IN2_ is the impedance observed from the signal input in the second-stage amplifier [24]. From the signal input, the inductor and capacitor (L_G3_ and C_G9_) are parallel to the inductor and resistor (L_G4_ and R_G6_) and capacitor (C_G10_). In addition, the output impedance of the second-stage amplifier (Z_OUT2_) illustrated in Figure 4b is obtained from Figure 2 as follows: Z_OUT2_ is the impedance observed from the signal output in the second-stage amplifier [24]. From the signal output, the inductor and capacitor (L_D3_ and C_D9_) are parallel to the inductor and resistor (L_D4_ and R_D2_) and capacitor (C_D10_). Z_IN2_ and Z_OUT2_ of the second stage are expressed by Equation (2).

(2) $$\begin{array}{c} Z_{IN2}=(j2\pi fL_{G3}+\frac{1}{j2\pi fC_{G9}})+\left\{(j2\pi fL_{G4}+R_{G6})∥\frac{1}{j2\pi fC_{G10}}\right\},\;\\ Z_{OUT2}=(j2\pi fL_{D3}+\frac{1}{j2\pi fC_{D9}})+\left\{(j2\pi fL_{D4}+R_{D2})∥\frac{1}{j2\pi fC_{D10}}\right\}. \end{array}$$

Based on the analysis above, the input and output poles (f_IN_ and f_OUT_) can be obtained using Z_IN1,2_ and Z_OUT1,2_. The f_IN_ and f_OUT_ mean the ratio of the amplifier response to the external input and output. The input and output poles (f_IN_ and f_OUT_) of the amplifier are as follows [62]:

(3) $$f_{IN}=\frac{1}{2\pi }\times \frac{1}{Z_{IN}[C_{GS}+(1+g_{m}Z_{OUT})C_{GD}]}\mathrm{and}\;f_{OUT}=\frac{1}{2\pi }\times \frac{1}{Z_{OUT}(C_{DS}+C_{GD})},$$

where g_m_ represents the transconductance and C_GS_, C_GD_, and C_DS_ are parasitic capacitances of the power MOSFET.

The gain equations (OUT_1_/IN_1_ and OUT_2_/IN_2_) are obtained as follows, and can be obtained through Equations (1)–(3). Finally, a single-ended output of the two-stage amplifier can be obtained [24],

(4) $$\frac{OUT_{1}}{IN_{1}}=\frac{-g_{m}Z_{OUT1}}{\left(1+\frac{j2\pi f}{f_{IN1}})(1+\frac{j2\pi f}{f_{OUT1}}\right)}\;and\;\frac{OUT_{2}}{IN_{2}}=\frac{-g_{m}Z_{OUT2}}{\left(1+\frac{j2\pi f}{f_{IN2}})(1+\frac{j2\pi f}{f_{OUT2}}\right)},$$

where IN_1_ and OUT_1_ are the first-stage amplifier input and output, respectively. IN_2_ and OUT_2_ are the second-stage amplifier input and output, respectively. f_IN1_ and f_OUT1_ are the input and output poles of the first-stage amplifier, respectively. f_IN2_ and f_OUT2_ are the input and output poles of the second-stage amplifier, respectively.

In conclusion, the final output and gain of the single-ended class-B amplifier (V_OUT_ and Gain) were calculated as,

(5) $$V_{OUT}=Gain\times IN_{1},$$

(6) $$Gain\;=\frac{OUT_{1}}{IN_{1}}\times \frac{OUT_{2}}{IN_{2}}.$$

### 2.3. Equivalent Circuit Analysis of Post-Voltage-Boost Circuit

**Figure 5:**
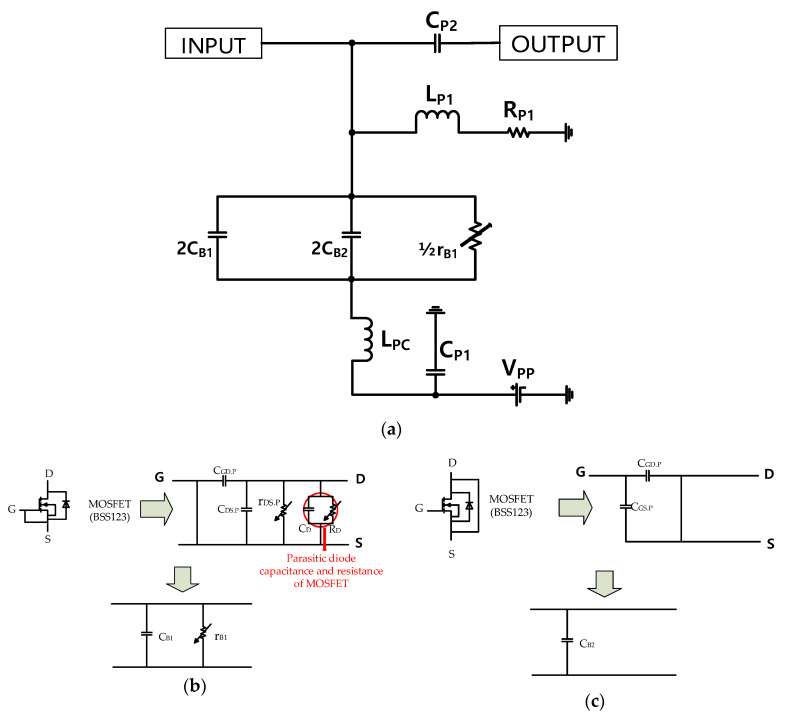
(**a**) Equivalent circuit of the post-voltage-boost circuit; (**b**) equivalent circuit of the gate-source metal-oxide-semiconductor field-effect transistor (MOSFET); and (**c**) equivalent circuit of the drain-source MOSFET.

Figure 5a shows the equivalent circuit model of the post-voltage-boost circuit, as shown in Figure 3. Each MOSFET in Figure 3 has two pairs of gate-source or drain-source connected transistors. The gate-source and drain-source connected MOSFETs are not working as active components, but they are working as passive components, which can be biased by DC voltages [28]. As shown in Figure 3, there is BSS123, which is an N-channel enhancement mode field effect transistor with parasitic diode. The gate-source connected transistor functions as a C_B1_ (capacitance combination of C_GD.P_, C_DS.P_, and C_D_) and r_B1_ (resistance combination of r_DS.P_ and R_D_) according to the supplied bias signals (V_pp_) [41]. The drain-source connected transistor functions as a C_B2_ (capacitance combination of C_GD.P_, C_GS.P_, and C_D_) according to the supplied DC bias signal (V_PP_) [53]. The values of r_B1_ change with the applied bias voltage; hence, they can affect the output amplitudes. The equivalent circuit of the gate-source or drain-source MOSFET are illustrated in Figure 5b,c. The C_GD.P_, C_DS.P_, and C_GS.P_ are the parasitic capacitances, and r_DS.P_ is the internal resistance of the MOSFET (BSS123). Moreover, C_D_ and R_D_ are the parasitic capacitance and resistance in the parasitic diode equivalent circuits of MOSFET (BSS123). In addition, the harmonic signal ranges are filtered by the resonance filters of C_P2_, L_P1_, and R_P1_. As shown in Figure 3, the post-voltage-boost circuit’s input port (INPUT) is connected to the amplifier’s output port (OUTPUT).

To obtain the final output of the post-voltage-boost circuit, we need to determine the impedance of the post-voltage-boost circuit. The impedance of the post-voltage-boost circuit (Z_P_) shown in Figure 5 is presented below. In the circuit of the post-voltage-boost circuit, the choke inductor (L_PC_) is connected in series with two pairs of resistors and capacitors (r_B1_ and C_B1,2_) connected in parallel, and it is also connected in parallel with the inductor and resistor (L_P1_ and R_P1_) connected in series. In addition, it is connected in parallel with a capacitor (C_P2_),

(7) $$Z_{P}=\left\{j2\pi fL_{PC}+2\frac{r_{B1}\times C_{B1}}{r_{B1}\times C_{B1}+j4\pi f\times C_{B1}\times C_{B2}}\right\}∥(j2\pi fL_{P1}+R_{P1})∥\frac{1}{j2\pi fC_{P2}}.$$

### 2.4. Equivalent Circuit Analysis of Class-B Amplifier and Post-Voltage-Boost Circuit

The poles (f_IN_ and f_OUT_) were obtained through the impedance of the Z_IN_ and Z_OUT_ of the class-B amplifier and the post-voltage-boost circuit. Z_IN_ is the same as using only the class-B amplifier, but Z_OUT2_ is different. The second-stage class-B amplifier Z_OUT2_ and the post-voltage-boost circuit impedance Z_P_ are applied. Therefore, the input pole (f_IN_) is the same as when only the class-B amplifier is used, and the pole for Z_OUT2_ of the class-B amplifier including Z_P_ is as follows. It can be obtained through Equations (1), (2), and (7),

(8) $$f_{IN}=\frac{1}{2\pi }\times \frac{1}{Z_{IN}[C_{GS}+(1+g_{m}Z_{OUT})C_{GD}]}\mathrm{and}f_{OUT.P}=\frac{1}{2\pi }\times \frac{1}{\left(Z_{OUT}∥Z_{P})(C_{DS}+C_{GD}\right)}.$$

where C_GS_, C_GD_, and C_DS_ are parasitic capacitors of the power MOSFET (PD57018-E).

The output of the amplifier and the post-voltage-boost circuit is calculated using the impedance of the post-voltage-boost circuit (Z_P_). Using Equations (1)–(3), (7), and (8), the gains of the first-stage amplifier and second-stage amplifier with post-voltage-boost circuit are calculated as follows [24]:

(9) $$\frac{OUT_{1}}{IN_{1}}=\frac{-g_{m}Z_{OUT1}}{\left(1+\frac{j2\pi f}{f_{IN1}})(1+\frac{j2\pi f}{f_{OUT1}}\right)}and\;\frac{OUT_{2.p}}{IN_{2.p}}=\frac{-g_{m}(Z_{OUT2}∥Z_{P})}{\left(1+\frac{j2\pi f}{f_{IN2}})(1+\frac{j2\pi f}{f_{OUT.P}}\right)},$$

where OUT_1_/IN_1_ is the gain of the first-stage amplifier, IN_2.p_ and OUT_2.p_ are the input and output of the post-voltage-boost circuit, respectively, and OUT_2.p_/IN_2.p_ is the gain of the second-stage of the class-B amplifier with a post-voltage-boost circuit. f_OUT2.P_ is the output pole of the second-stage amplifier with post-voltage-boost circuit.

As shown in Equation (7), the impedance of the post-voltage-boost circuit (Z_P_) is related to the output impedances (Z_OUT2_) and parasitic impedances (C_GS_, C_GD_, and C_DS_) of the second-stage amplifier in Equation (2) because the inductance (L_PC_) with variable resistances (r_B1_) depending on the applied DC voltage (V_PP_) and parasitic capacitances (C_B1_ and C_B2_) of the MOSFET (BSS123) in the post-voltage-boost circuit could influence the inductance (L_D3_ and L_D4_) and capacitance (C_D9_ and C_D10_) in the output impedance (Z_OUT2_) with parasitic capacitances (C_GS_, C_GD_, C_DS_, and C_GD_) of the MOSFET (PD57018) in the second-stage amplifier. Consequently, the impedance, Z_P_ of the post-voltage-boost circuit with a DC voltage applied affects the impedance Z_OUT2_ of the second-stage amplifier; then, the gain of the second-stage amplifier with a post-voltage-boost circuit (Gain._P_) is affected by the impedance Z_P_. Therefore, the final output (V_OUT.P_) of the amplifier with a post-voltage-boost circuit could be changeable depending on the applied conditions. From Equations (7) and (8), the final output (V_OUT.P_) and total gain (Gain._P_) of the class-B amplifier and post-voltage-boost circuit are provided below [24]. Therefore, the final output of the amplifier with a post-voltage-boost circuit could be changeable depending on the applied conditions,

(10) $$V_{OUT.P}=Gain\times IN_{1},$$

(11) $$Gain.p\;=\frac{OUT_{1}}{IN_{1}}\times \frac{OUT_{2.p}}{IN_{2.p}}\;.$$

**Figure 6:**
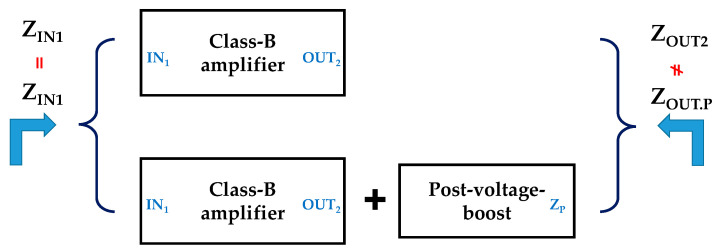
Difference between amplifier only and amplifier with post-voltage-boost circuit.

When analyzing the impedance of the designed class-B amplifier and class-B amplifier with a post-voltage-boost circuit, the impedance at the input side is the same. However, the impedance of the output side is different from the impedance of the class-B amplifier as shown in the second stage and the impedance as shown in the post-voltage-boost circuit. The post-voltage-boost circuit can compensate the unnecessary parasitic impedances of the class-B amplifier to show the gain effect improvement. Figure 6 shows the description of the proposed concept.

Both Z_IN1_ of the only class-B amplifier and the class-B amplifier with post-voltage-boost circuit are the same, but the transmission function and final output functions are different because Z_OUT2_ is different owing to the impedance (Z_P_) of the amplifier’s post-voltage-boost circuit. Thus, we can compare the final output of both circuits as shown in Equation (12). The input and output poles of both circuits can be compared through Equations (3) and (8).

(12) $$\begin{array}{l} V_{OUT}=Gain\times IN_{1}\\ \neq V_{OUT.P}=Gain.p\times IN_{1}, \end{array}$$

where V_OUT_ is the final output of the amplifier, V_OUT.P_ is the final output of the amplifier with a post-voltage-boost circuit.

The power MOSFETs used in the analysis have variable and inaccurate performance according to the different temperatures and high DC voltage levels [57,63]. There are always errors between the experimental results and theoretical analysis under a high-voltage and high-current test environment [64]. The signal distortions that are present during the amplifier design in a high-voltage and high-current environment make it difficult to accurately predict the performance of the amplifiers [65,66,67,68]. In addition, the output performance of the amplifier in the experiments may fluctuate widely owing to external environmental factors such as the heat and power level parameters [41,57,69]. Consequently, the theoretical analysis of the amplifier and post-voltage-boost circuit should be verified using actual measurement data. These calculated and simulated data are different with measurement data due to inaccurate simulation library data.

## 3. Results

**Figure 7:**
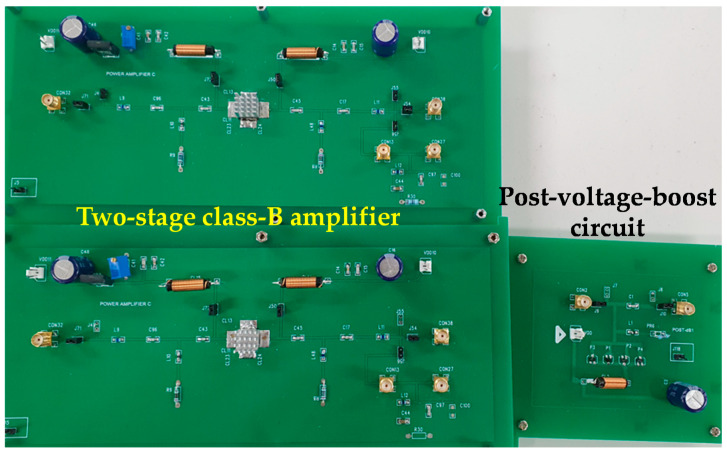
Printed circuit board (PCB) of the class-B amplifier and post-voltage-boost circuit.

Figure 7 shows a printed circuit board (PCB) of the class-B amplifier and post-voltage-boost circuits. Power resistors, high-power choke inductors, and electrolytic capacitors have been used to ensure safe operation under high-voltage environments [49].

### 3.1. Performance Analysis

**Figure 8:**
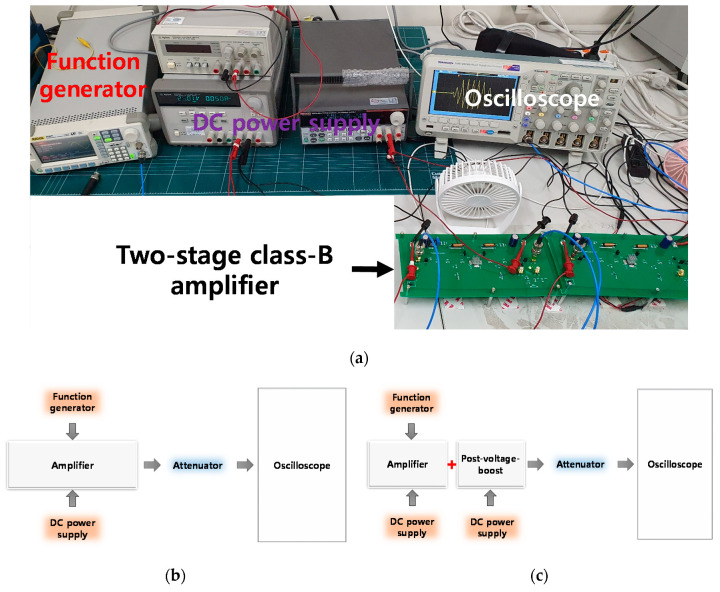
(**a**) Experimental measurement environment; (**b**) block diagram of how the performance of a class-B amplifier is measured; (**c**) block diagram of how the performance of a class-B amplifier with post-voltage-boost circuit is measured.

Figure 8a shows the experimental measurement environment. A function generator, DC power supply, and oscilloscope were used for the measurement. Figure 8b,c show the block diagrams of the experimental methods for measuring the performance of a class-B amplifier only and class-B amplifier with post-voltage-boost circuits. It was set to generate 5-cycle sine wave input voltages from the function generator. The DC bias voltage was applied to the amplifier and a post-voltage-boost circuit via a DC power supply. In addition, overvoltage damage in the oscilloscope was prevented using a 50-W and 40-dB attenuator. Further, the external coolers and heat sinks were used to minimize the heat generation effects from the power MOSFET and other electronic devices [70,71,72].

**Figure 9:**
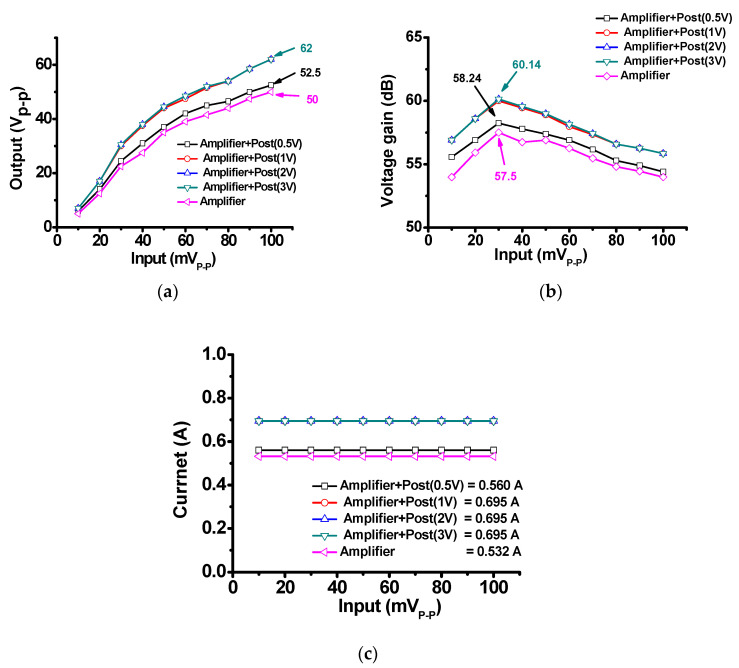
(**a**) Input voltage versus output voltage; (**b**) input voltage versus voltage gain; and (**c**) current consumption of only the amplifier and the amplifier with post-voltage-boost circuit according to different DC input voltages.

Figure 9 shows the measured performance of the designed class-B amplifier and class-B amplifier with post-voltage-boost circuits. The input voltages range from 10 mV_P-P_ to 100 mV_P-P_, and the DC voltages of the post-voltage-boost circuit were 0.5, 1, 2, and 3 V. Figure 9a shows the variation of the input signal (mV_P-P_) with the output signal (V_P-P_) of the designed class-B amplifier and class-B amplifier with post-voltage-boost circuits. The amplifier represents the two-stage class-B amplifier. Amplifier + Post (0.5 V), Amplifier + Post (1 V), Amplifier + Post (2 V), and Amplifier + Post (3 V) represent the two-stage class-B amplifier with a post-voltage-boost circuit with a 0.5, 1, 2, and 3 V DC bias voltage applied. The threshold voltage of MOSFET (BSS123) is 0.8 V. As the DC bias voltage higher than threshold voltage is applied to the MOSFET, the MOSFET has similar parasitic gate-source, gate-drain, and drain-source capacitance values with drain-source resistance such that there should be performance differences between Amplifier + Post (0.5 V) or Amplifier + Post (1 V), Amplifier + Post (2 V), and Amplifier + Post (3 V). The DC bias voltage (V_PP_) affects the parasitic diode resistances (r_D1_ and r_D2_) in the post-voltage-boost such that variable resistances affect the output voltage of the amplifier.

Based on the measured output signals of the amplifiers (Figure 9a), the output signal amplitude was obtained when using the amplifier with the post-voltage-boost (1, 2, and 3 V) circuit (62 V_P-P_) was higher than that obtained when using only the amplifier (50 V_P-P_) with a 100-mV_P-P_ input. Thus, a higher output signal can be obtained when a post-voltage-boost circuit is incorporated into the design. In addition, when the 0.5-V DC voltage of the post-voltage-boost circuit was applied, a slightly higher output signal (Amplifier + Post (0.5 V)) was obtained, i.e., 52.5 V_P-P_ at 100 mV_P-P_. Figure 9b shows the variation of the input signal (mV_P-P_) with the voltage gain (dB) of the designed class-B amplifier and class-B amplifier with post-voltage-boost circuits. The maximum voltage gain obtained when using the amplifier with a post-voltage-boost (1, 2, and 3 V) circuit (60.14 dB) was higher than that obtained when using only the amplifier (57.5 dB) when the input signal is 30 mV_P-P_. The voltage gain achieved when using the amplifier with a post-voltage-boost (1, 2, and 3 V) circuit (55.84 dB) was higher than that achieved when using only the amplifier (53.97 dB) when the input signal is 100 mV_P-P_. Figure 9c shows the current consumption of the designed class-B amplifier and class-B amplifier with post-voltage-boost circuits. When DC bias voltage less than the threshold voltage is applied to the MOSFET, the post-voltage-boost circuit can be start to work. When the DC bias voltage is higher than the threshold voltage of the MOSFET, the post-voltage-boost circuit can be fully working with other components. According to the different DC bias voltage, the variable resistance (r_B1_) in the post-voltage-boost circuit could affect the DC current consumption such that the DC current consumption has been changed, as shown in Figure 9c. When using only the amplifier, the DC current according to the input is 0.532 A, and when using the post-voltage-boost (0.5 V) circuit, the DC current according to the input is 0.56 A. When using a post-voltage-boost (1, 2, and 3 V) circuit, the DC current according to the input was 0.695 A. The current consumption increased a little bit when the post-voltage-boost circuit worked. The current consumptions when using the amplifier-only circuit and using the amplifier with post-voltage-boost (1~3 V) circuit were 0.532 and 0.695 A. The measured output amplitude values in Figure 9a are summarized in Table 1. The output voltages and voltage gain of the amplifier with a post-voltage-boost circuit have higher output values than those obtained when using only the amplifier.

**Figure 10:**
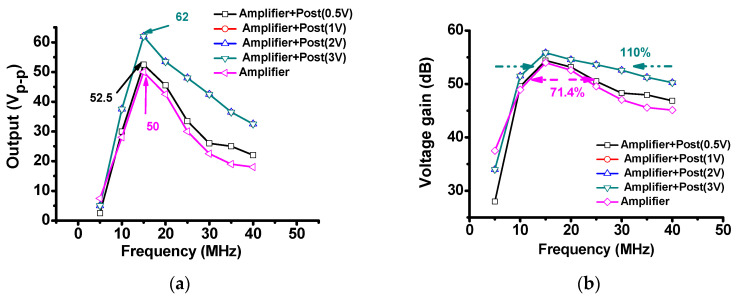

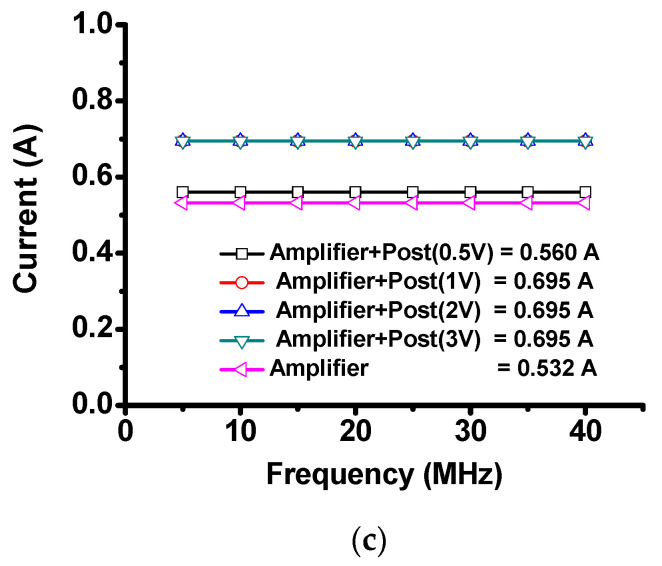
(**a**) Output voltage versus frequency; (**b**) voltage gain versus frequency; and (**c**) current consumption versus frequency for only the amplifier and amplifier with post-voltage-boost circuits.

Figure 10a shows the variation of the output voltage with the frequency range of the amplifiers when a 100-mV_P-P_ input voltage was applied. As shown in Figure 10a, when a post-voltage-boost circuit was used together, the highest output was measured at a center frequency of 15 MHz and an input of 100 mV_P-P_. The output amplitude obtained when using the amplifier with a post-voltage-boost circuit (62 V_P-P_) was higher than that obtained when using only the amplifier (50 V_P-P_). Figure 10b shows the voltage gain over the frequency range of the amplifiers. When only the amplifier was used, the −3 dB bandwidth is 71.4% at the 15-MHz center frequency. When the amplifier and post-voltage-boost circuits were used together, the −3 dB bandwidth was 110% at the center frequency of 15 MHz. Figure 10c shows the measured current consumption of the amplifiers for different frequency ranges. The measured output amplitudes in Figure 10a are summarized in Table 2. The amplifier with the post-voltage-boost circuit had higher output values and bandwidth compared to only the amplifier. However, the DC voltages of 1, 2, and 3 V for the post-voltage-boost circuit have similar DC current consumption values.

Since the mobile ultrasound system was required to use battery all the time, the power consumption of the amplifier, which was one of the most consuming power sources in the system, needs to be considered in the design level. The developed amplifier with post-voltage-boost circuit was used in the mobile ultrasound system such that the power consumption needs to be measured [41]. The power-added efficiency (PAE) equation is shown below

(13) $$\;PAE\;(\%)\;=\;\frac{P_{OUT}-P_{IN}}{DC\;power}\;\times \;100\%.$$

where P_OUT_ and P_IN_ are the output power and input power of the amplifier, and DC power is the DC power consumption of the amplifier.

**Figure 11:**
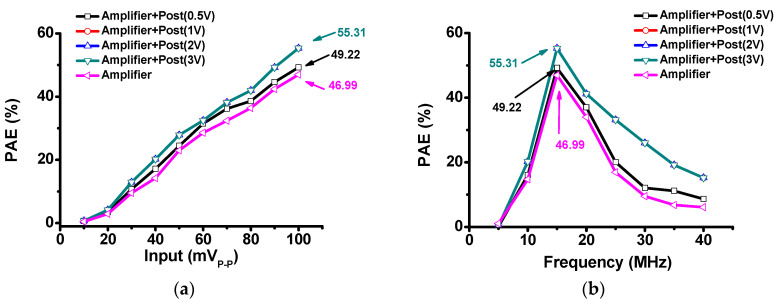
(**a**) Power-added efficiency (PAE) versus input voltage; (**b**) PAE versus frequency.

Figure 11 shows the power-added efficiency (PAE) comparison of only the amplifier and amplifier with the post-voltage-boost circuits according to the input voltage and frequency. In Figure 11a, the PAE of the only class-B amplifier was measured to be 46.99% at 100 mV input. When using the amplifier and post-voltage-boost circuit (1, 2, and 3 V), the PAE was increased to be 55.31% at 100 mV input. In Figure 11b, the class-B amplifier was measured to be 46.99% at 15 MHz input. The PAE when using the amplifier and post-voltage-boost circuit (1, 2, and 3 V) was increased to be 55.31% at 15 MHz. As shown above, the current consumption when post-voltage-boost circuit was a little bit increased (0.532 A to 0.560 A); however, the output voltage or gain when using the post-voltage-boost circuit was improved (50 to 62 V or 57.5 to 60.14 dB). The lower the DC consumption and the higher the final output, the higher the efficiency or PAE could be obtained. When using the post-voltage-boost circuit, the DC consumption increased, but the output increased more, thus the efficiency (PAE) of the amplifier with post-voltage-boost circuit was improved.

**Figure 12:**
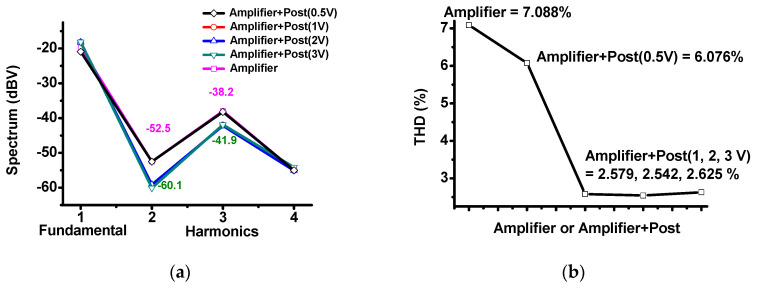
(**a**) Fast-Fourier-transform (FFT) harmonic spectrum of only the amplifier and amplifier with post-voltage-boost circuits; (**b**) total harmonic distortion (THD) of only the amplifier and amplifier with post-voltage-boost circuits.

Figure 12 shows the fast-Fourier-transform (FFT) harmonic spectrum data and total harmonic distortion (THD) of only the amplifier and amplifier with post-voltage-boost circuits when there is a 100-mV input at 15 MHz. Figure 10a shows the spectrum data of only the amplifier or amplifier with post-voltage-boost circuits. The output signal FFT of the amplifier was −20.9 dB at the fundamental frequency (15 MHz), −52.5 dB at the second harmonic (30 MHz), −38.2 dB at the third harmonic (45 MHz), and −55 dB at the fourth harmonic (60 MHz). However, it can be observed that the harmonics were slightly reduced when the amplifier and the post-voltage-boost circuit were used together. When the 3-V DC value of the post-voltage-boost circuit was applied, the output signal FFT was −18.1 dB at the fundamental frequency (15 MHz), −60.1 dB at the second harmonic (30 MHz), −41.9 dB at the third harmonic (45 MHz), and −54.1 dB at the fourth harmonic (60 MHz). Figure 10b shows the THD of only the amplifier and amplifier with post-linearizer circuitry. The THD (%) values were calculated using Equation (14). The THD (%) of the amplifier was 7.088%. The THD (%) of the amplifier with a post-voltage-boost circuit and a 0.5-V DC voltage was 6.076%. The THD (%) of the amplifier with a post-voltage-boost circuit with DC voltages of 1, 2, and 3 V were 2.579, 2.542, and 2.625%, respectively. It can be confirmed that when the post-voltage-boost circuit was used, the second and third harmonic values and THD (%) decreased,

(14) $$THD\;(\%)=\frac{\sqrt{\mathrm{Sec}ond.Harmonic^{2}+Third.Harmonic^{2}+Fourth.Harmonic^{2}}}{Fundamental}\times 100\%.$$

The comparison data with other work present in the literature is shown in Table 3.

### 3.2. Pulse-Echo Analysis

**Figure 13:**
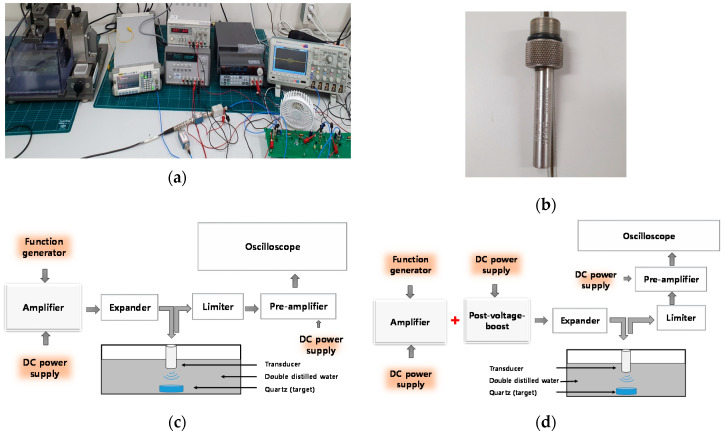
(**a**) Experimental measurement environment; (**b**) tested ultrasound transducer; (**c**) pulse-echo signal measurement setup for the amplifier; (**d**) pulse-echo signal measurement setup for the amplifier with post-voltage-boost circuit.

The pulse-echo experiment is a basic indicator for performance tests such as ultrasonic systems or ultrasonic transducer configurations [4,9], and their performance can be estimated by measuring echo amplitudes, pulse-widths, harmonics, etc. [74,75]. Figure 13a shows a picture of the experimental measurement environment. The transducer of the pulse-echo test was a 15-MHz 1/4” diameter ultrasonic immersion transducer (I21504T) provided by Olympus (Shinjuku-ku, Tokyo, Japan), as shown in Figure 13b. Figure 13c,d show the block diagram of the experimental methods employed for performing the pulse-echo test. The input signal of the amplifier was a 5-cycle, 100-mV_P-P_ sinusoidal waveform at 15 MHz. The expander used in the experiment had a pair of diodes, which were used to reduce the ring-down signal [38,76]. In the limiter used in the experiment, a pair of diodes were connected in parallel with a resistor, and it was used to reduce the high-voltage signal to protect the oscilloscope and pre-amplifier [35,77,78]. The measured echo signal that was reflected through the target (Quartz) from the transducer in double-distilled water was amplified by only the amplifier and amplifier with a post-voltage-boost circuit. The quartz can reflect acoustic signals completely back, such that this target can be useful to estimate the developed ultrasound components.

**Figure 14:**
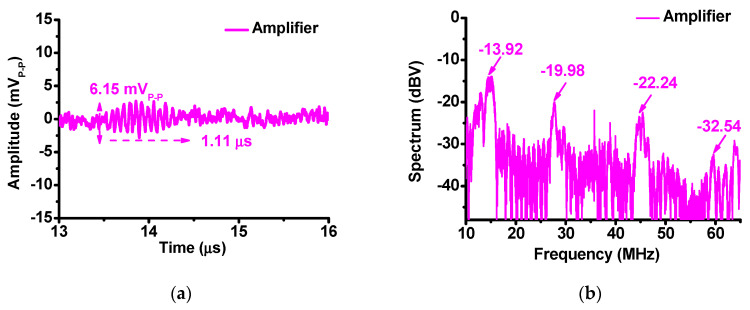
(**a**) Echo signal of amplifier; (**b**) FFT spectrum of amplifier.

Figure 14a,b show the echo signal and FFT spectrum of only the amplifier when using a 15-MHz ultrasound transducer. The amplitude and pulse-width of the echo signal were 6.15 mV_P-P_ and 1.11 μs, respectively. The FFT of the echo signal was −13.92 dB at the fundamental frequency (15 MHz), −19.98 dB at the second harmonic (30 MHz), −22.24 dB at the third harmonic (45 MHz), and −32.54 dB at the fourth harmonic (60 MHz).

**Figure 15:**
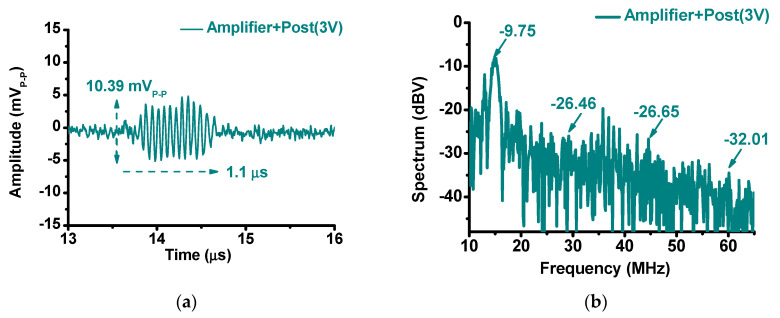
(**a**) Echo signal and (**b**) FFT of amplifier and post-voltage-boost circuit with tested ultrasonic transducer.

Figure 15a,b show the echo signal and FFT spectrum of the amplifier with the post-voltage-boost circuit (3 V) using a 15-MHz ultrasound transducer. The amplitude and pulse width of the echo signal were 10.39 mV_P-P_ and 1.11 µs, respectively. The FFT of the echo signal was −9.75 dB at the fundamental frequency (15 MHz), −26.46 dB at the second harmonic (30 MHz), −26.65 dB at the third harmonic (45 MHz), and −32.01 dB at the fourth harmonic (60 MHz). By comparing the results in Figure 12 and Figure 13, we can confirm that the use of the amplifier and the post-voltage-boost circuit had a positive effect on the echo signal and FFT spectrum. When using the post-voltage-boost circuit, the gain of the amplifier increased, so the pulse-echo signal increased accordingly.

**Figure 16:**
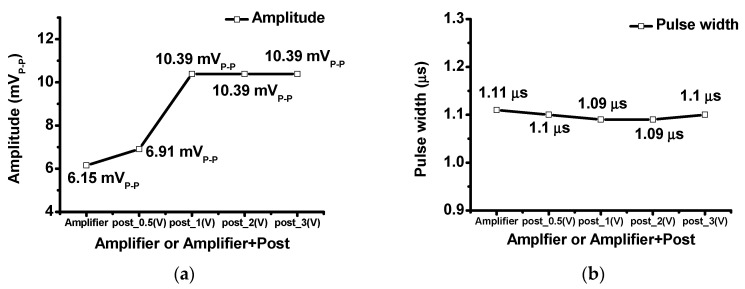
(**a**) Echo signal and (**b**) echo signal comparisons when using only the amplifier and amplifier with post-voltage-boost circuits.

Figure 16a,b show the comparison of the echo amplitudes and pulse widths when using only the amplifier and amplifier with post-voltage-boost circuits. The echo amplitude of the amplifier with a post-voltage-boost circuit had a larger amplitude than that of only the amplifier, but the pulse-width of only the amplifier and the amplifier with the post-voltage-boost circuit was approximately 1.1 µs. The echo signal amplitude obtained when using only the amplifier was 6.15 mV_P-P_, the echo signal of the amplifier with the post-voltage-boost circuit (0.5 V) was 6.91 mV_P-P_, and the echo signal of the amplifier with the post-voltage-boost circuit (1, 2, and 3 V) was 10.39 mV_P -P_.

The impedance, Z_P_, of the post-voltage-boost circuit with a DC bias voltage applied affects the impedance, Z_OUT2_ of the second-stage amplifier; then, the output voltage of the second-stage amplifier with a post-voltage-boost circuit is affected by the impedance, Z_P_. When using the post-voltage-boost circuit, the output voltage of the amplifier was increased from 50 to 62 V_P-P_. As shown in Figure 14a and Figure 15a, the echo amplitude generated from the piezoelectric transducer was increased from 6.15 mV_p-p_ to 10.39 mV_p-p_, accordingly. The resonant filter architecture in the post-voltage-boost circuit could help reduce the second and third harmonic distortions (HD2 = −60.1dB and HD3 = −41.9 dB) such that the echo spectrum has a little bit lower second and third harmonic distortions (HD2 = −26.46 dB and HD3 = −26.65 dB) as shown in Figure 14b and Figure 15b.

## 4. Conclusions

In mobile ultrasound systems, limited battery capacity, low DC current capacity, and the number of electronic channels significantly deteriorate the sensitivity performance of piezoelectric transducers compared to piezoelectric transducers used in bench-top ultrasound systems. Therefore, it is challenging to design efficient electronic devices owing to several bottlenecks such as current consumption, power supply levels, and size constraints (e.g., compact cellular phones). Even the performance of the piezoelectric transducers varies with respect to the frequency and voltage levels, and this is undesirable when aiming to achieve stable electrical performance for such high-frequency (15 MHz) piezoelectric transducers. Consequently, few studies have focused on developing electronic devices to improve the performance of piezoelectric transducers. The post-type circuit can easily vary the performance of piezoelectric transducers because the circuit is located between the amplifier and piezoelectric transducer. Therefore, it is beneficial to have a single-ended class-B amplifier integrated with a post-voltage-boost circuit to increase the amplitude of the piezoelectric transducer for mobile ultrasound applications. In our proposed method, the impedance of the post-voltage-boost circuit with a DC bias voltage applied affects the impedance of the second-stage amplifier; then, the output voltage of the second-stage amplifier with a post-voltage-boost circuit is affected by the impedance of the post-voltage-boost circuit. When using the post-voltage-boost circuit, the output voltage of the amplifier was improved accordingly.

The measured voltage amplitude of the class-B amplifier with the post-voltage-boost circuit (62 V_P-P_) is higher than that for only the class-B amplifier (50 V_P-P_) at 15 MHz and with a 100-mV_P-P_ input. In the pulse-echo measurement test, the echo signal with the class-B amplifier with post-voltage-boost circuit (10.39 mV_P-P_) was also higher than that with only the class-B amplifier (6.15 mV_P-P_). The DC current consumption was 0.532 A when using only the amplifier. The DC current consumption were 0.56 A or 0.695 A when using the post-voltage-boost circuit, and had a difference of up to 0.163 A. The PAE was 46.99% when using only the amplifier. The PAE were 49.22 or 55.31% when using the amplifier with post-voltage-boost circuit together. Therefore, this designed post-voltage-boost circuit could be more efficient for piezoelectric transducers with lower sensitivity used for mobile ultrasound applications.

The proposed post-voltage-boost circuit scheme may be simpler compared to the pre-distortion scheme using ADC, DAC, memory, and FPGA. Most mobile ultrasound systems, such as mobile imaging machines, use multi-channel piezoelectric transducers with limited sizes to boost the amplifier performance, and so a compact design is essential. The proposed post-voltage-boost circuit needs to estimate several mathematical approaches and appropriate design values for the gain increasing method, but it can be used with only a few passive and active components. Intravascular ultrasound machines, which require a high sensitivity owing to the small sizes of the piezoelectric components, could be useful in increasing the echo signal amplitudes, thus improving the signal sensitivity.

## Figures and Tables

**Table 1:** Measured output amplitudes in Figure 9a.

Input [mV_P-P_]	Amplifier [V_P-P_]	Amplifier with Post (0.5 V) [V_P-P_]	Amplifier with Post (1 V) [V_P-P_]	Amplifier with Post (2 V) [V_P-P_]	Amplifier with Post (3 V) [V_P-P_]
10	5	6	7	7	7
20	12.5	14	17	17	17
30	22.5	24.5	30	30.5	30.5
40	27.5	31	37.5	38	38
50	35	37	44	44.5	44.5
60	39	42	47.5	48.5	48.5
70	41.5	45	51.5	52	52
80	44	46.5	54	54	54
90	47.5	50	58.5	58.5	58.5
100	50	52.5	62	62	62

**Table 2:** Measured output amplitudes in Figure 10a.

Frequency [MHz]	Amplifier [V_P-P_]	Amplifier with Post (0.5 V) [V_P-P_]	Amplifier with Post (1 V) [V_P-P_]	Amplifier with Post (2 V) [V_P-P_]	Amplifier with Post (3 V) [V_P-P_]
5	7.5	2.5	5	5	5
10	28	30	37.5	37.5	37.5
15	50	52.5	62	62	62
20	42.5	45.5	53.5	53.5	53.5
25	30	33.5	48	48	48
30	22.5	26	42.5	42.5	42.5
35	19	25	36.5	36.5	36.5
40	18	22	32.5	32.5	32.5

**Table 3:** Comparison data with other work in the literature.

	This Work	[30]	[73]	[34]
Mode	Class-B	Class-AB	Class-D	Class-A
Frequency	15 MHz	5 MHz	10 kHz	10 MHz
Output	62 V_P-P_	180 V_P-P_	2 kW	-
Gain	60.14 dB	-	-	15.6 dB
PAE	55.31%	44%	-	-
Harmonic distortion	HD2 = −60.1 dB	HD2 = −61.28 dB		HD2 = −8.94 dB
	HD3 = −41.9 dB	HD3 = −56.17 dB	-	HD3 = −10.01 dB

HD2 and HD3 represent second-order and third-order harmonic distortions, respectively.
